# New Advances in Antenna Design toward Wearable Devices Based on Nanomaterials

**DOI:** 10.3390/bios14010035

**Published:** 2024-01-10

**Authors:** Chunge Wang, Ning Zhang, Chen Liu, Bangbang Ma, Keke Zhang, Rongzhi Li, Qianqian Wang, Sheng Zhang

**Affiliations:** 1 School of Mechanical and Energy Engineering, NingboTech University, Ningbo 315100, China; wangchunge@nit.zju.edu.cn (C.W.); ydzn97@stumail.ysu.edu.cn (N.Z.); zkk@stumail.ysu.edu.cn (K.Z.); 2 Key Laboratory of Advanced Forging & Stamping Technology and Science, Yanshan University, Ministry of Education of China, Qinhuangdao 066004, China; 3 Ningbo Innovation Center, Zhejiang University, Ningbo 315100, China; liuchen@nit.zju.edu.cn; 4 Faculty of Science and Engineering, University of Nottingham Ningbo, Ningbo 315100, China; 5 Ningbo L.K. Technology Co., Ltd., Ningbo 315100, China; bond@stumail.ysu.edu.cn; 6 Beijing Advanced Innovation Center of Materials Genome Engineering, State Key Laboratory for Advanced Metals and Materials, University of Science and Technology Beijing, Beijing 100083, China; d202310599@xs.ustb.edu.cn

**Keywords:** wearable device, wearable antenna, nanomaterials, carbon nanotubes, silver nanowires, graphene

## Abstract

Wearable antennas have recently garnered significant attention due to their attractive properties and potential for creating lightweight, compact, low-cost, and multifunctional wireless communication systems. With the breakthrough progress in nanomaterial research, the use of lightweight materials has paved the way for the widespread application of wearable antennas. Compared with traditional metallic materials like copper, aluminum, and nickel, nanoscale entities including zero-dimensional (0-D) nanoparticles, one-dimensional (1-D) nanofibers or nanotubes, and two-dimensional (2-D) nanosheets exhibit superior physical, electrochemical, and performance characteristics. These properties significantly enhance the potential for constructing durable electronic composites. Furthermore, the antenna exhibits compact size and high deformation stability, accompanied by greater portability and wear resistance, owing to the high surface-to-volume ratio and flexibility of nanomaterials. This paper systematically discusses the latest advancements in wearable antennas based on 0-D, 1-D, and 2-D nanomaterials, providing a comprehensive overview of their development and future prospects in the field.

## 1. Introduction

The application of wearable devices in daily life is becoming increasingly extensive [1,2]. These devices are not restricted to wristwatches, exercise shoes, and virtual reality glasses, and include other medical devices as well [3]. The wearable industry is expected to grow to USD 54 billion by 2023 [4]. Antennas are one of the essential aspects of wearable gadgets as they lead to a portable link for the overall effectiveness of the devices [5]. For better application in wearable systems, several usage specifications of the antenna must be considered, such as small size [6], low weight [7], power consumption, and flexible structure [8]. In addition, the impact of the wearable antenna on the host body during use and the possible significant loss of efficiency and reliability due to antenna deformation should be considered [9]. Therefore, when designing wearable antennas, several aspects need special consideration. The first is to deal with the deformation of the antenna when it is worn, as this can significantly degrade transmission performance [10]. The second is to take into account the coupling between the wearable antenna and the human body to prevent further performance degradation. Finally, the robustness of the antenna under various conditions, such as humidity, temperature, and distance between the body and the antenna, must be considered [11]. In addition, besides the basic parameters of the antenna, high electrical and mechanical performance, low cost, light weight, low loss, flexibility, ease of wearing, and high resolution/precision in the fabrication method are also very important [12].

With the improvement in nanomaterial development methods and the development of integrated wearable devices, nanomaterials and nanocomposites have become the focus of finding new integration concepts to improve antenna performance, which also puts forward new requirements for materials [13]. These nanomaterials mainly include metal nanoparticles [14,15,16], carbon nanotubes (CNTs) [17], silver nanowires (AgNWs) [18], graphene [19], MXene inks [20], conductive polymers [21], etc. Research results show that the wearable antenna has significantly enhanced antenna parameters due to the addition of nanomaterials, e.g., bandwidth enhancement, gain improvement, size reduction, and cost reduction.

As zero-dimensional (0-D) nanoparticles, metal nanoparticles have exceptional conductivity and minuscule size, such as silver, gold, or copper, and can enhance the electromagnetic properties of wearable antennas. In addition, these nanoparticles are capable of increasing the antenna’s gain and efficiency, while also preserving its lightweight and flexible nature. For example, in high-precision positioning systems, metal nanoparticles demonstrate particularly effective performance [22].

CNTs, as one-dimensional (1-D) nanomaterials, are renowned for their exceptional mechanical strength and good electrical conductivity. In wearable antennas, CNTs can be employed to enhance the mechanical stability and durability of the antenna, while maintaining certain electromagnetic properties. For instance, in sports monitoring and health monitoring systems, CNTs are a guarantee of a combination of mechanical strength and flexibility [3]. AgNWs combine the exceptional electrical conductivity of silver with the flexibility of nanoscale dimensions. In wearable antennas, AgNWs are primarily used to enhance electromagnetic performance, particularly in terms of gain and bandwidth. AgNWs are especially suitable for applications requiring high-frequency broadband communication, including high-speed data transmission and high-resolution wireless sensor networks [23].

Graphene, a two-dimensional (2-D) carbon material, is renowned for its exceptional electromagnetic properties and mechanical strength. Specifically, graphene is most effective in scenarios where a combination of high mechanical strength and superior electromagnetic performance is required, e.g., in advanced health monitoring systems and complex environmental sensing applications [24].

**Figure 1:**
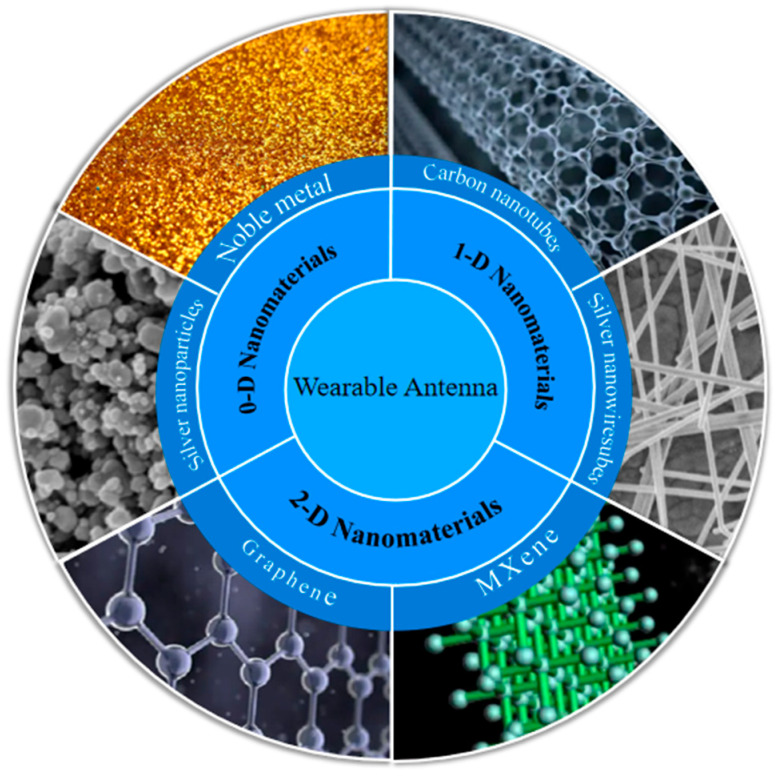
Nanomaterials are commonly used in wearable antennas.

The article classifies the nanomaterials for wearable antennas into three categories including 0-D, 1-D, and 2-D nanomaterials according to their dimensions, as shown in Figure 1, and summarizes the latest achievements of flexible wearable antennas in recent years. Moreover, the performance of the wearable antennas based on nanomaterials is analyzed. Finally, conclusions and future directions are given in this manuscript.

## 2. Wearable Antenna Parameters

Antennas play a very important role in wireless data transmission, and wearable antennas for human body communication are even more different from conventional antenna designs [25]. A wearable antenna, as an indispensable part of the radio frequency front-end in wearable devices, usually acts as an energy converter to receive and radiate radio waves, and its performance directly affects whether the human body’s wireless communication system can work normally. Therefore, the most intuitive tool to measure the performance of the antenna is the antenna parameters, which are often evaluated from the following three aspects: impedance matching, antenna pattern, and gain [26]. Impedance matching describes the appropriate degree of matching between the feeder and the antenna, which directly determines whether the feeder signal can be transmitted to the antenna through the feeder. The better the matching, the fewer reflected waves and the higher the feeding efficiency; conversely, the worse the impedance matching, the more serious the mismatch, and the lower the feeding efficiency [27]. In studies on wearable antennas, the degree of impedance matching is usually measured by the return loss (RL) or the voltage standing wave ratio (VSWR) of the transmission line. The RL represents the ratio of the power of the reflected wave to the incident wave on the feeder ($RL=P_{r}/P_{i}$) and is characterized by decibels (dBs). Usually, when the RL value is greater than 10 dB, the antenna is considered to have impedance matching in this frequency band [28]. The VSWR on an antenna refers to the ratio of the amplitude of the antinode voltage to the node voltage, that is, $VSER={\left|V\right|}_{max}/{\left|V\right|}_{min}$. It can be seen that when the VSWR approaches 1, the matching of the antenna system is the best, but when it approaches infinity, the matching is the worst, which is a state of total reflection. Usually, the VSWR of the antenna system in the project is required to be less than 2, that is, to meet the impedance matching [29]. The antenna pattern describes the radiation characteristics in free space and different azimuths and characterizes the relationship between the radiation field characteristics (field strength, phase, power density, gain, etc.) and the spatial angle. The pattern can directly reflect the directivity of the antenna. In detail, the narrower the lobe width of the pattern, the more concentrated the energy radiated by the antenna. In addition, the radiation characteristics of the antenna are usually characterized by a two-dimensional radiation pattern in the form of two vertically orthogonal planes, namely the H-plane and the E-plane. Gain describes the strength of the antenna’s radiating ability in a specific direction, usually referring to the direction of the maximum radiation intensity in space. It is mainly limited by the directivity coefficient D and the radiation efficiency *η*, and can be expressed by the following formula: G=η·D ($\eta =P_{rad}/P_{in}$, *η* represents the radiation efficiency, $P_{rad}$ is the power radiated by the antenna, and $P_{in}$ is the total power input to the antenna). It can be seen that when the directivity coefficient of the antenna is constant, the gain mainly depends on the efficiency.

## 3. Wearable Antenna Materials

In the past few years, major research breakthroughs in nanomaterials have enabled their widespread application in wearable antennas [30]. However, compared with copper, aluminum, nickel, and other metal materials, nanoparticles (0-D) [31], one-dimensional nanofibers or nanotubes (1-D) [32], and nanosheets (2-D) [33] show better physical, electrochemical, and performance properties and greater potential for building wear-resistant electronic composites [34]. In addition, the high surface-to-volume ratio and high flexibility of nanomaterials endow the antenna with small size and high deformation stability, making it more portable and wear-resistant [35,36].

### 3.1. 0-D Nanomaterial Structure Antenna

0-D nanostructured materials refer to materials that have dimensions at the nanoscale in all directions, primarily including quantum dots, nanoclusters, and nanocrystals. The main example of this part is to incorporate metal and metal compound nanoparticles into a polymer matrix or flexible film through specific technical means to better realize the physical properties of wearable antennae in terms of flexibility, adaptability, and electromechanical stretching [37]. Over the past few years, the demand for wearable devices has increased with the miniaturization of electronic systems. Therefore, the field of wearable devices has become the most attractive topic for researchers who have developed various small antennas with good performance [38]. However, for small wearable devices, it is one of the challenges of the current technology that the antenna can be operated at low frequencies without interfering with radiation efficiency and gain [39].

**Figure 2:**
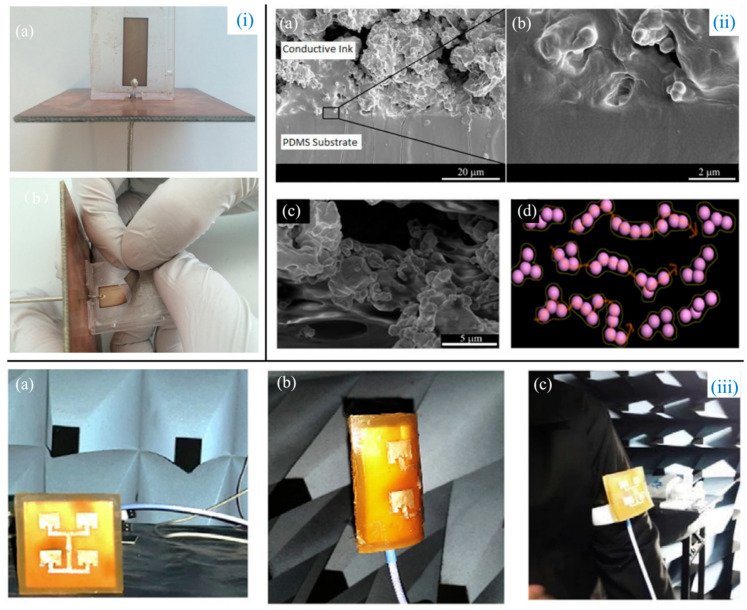
(**i**) (**a**) Photograph of the antenna produced using inkjet printing; (**b**) demonstration of the flexibility of inkjet-printed antenna [41]. (**ii**) (**a**,**b**) The cross-sectional and locally enlarged scanning electron microscope (SEM) images of conductive ink on a PDMS substrate; (**c**,**d**) SEM images and schematic representation of stages of silver particle realignment in polymer matrix during globular rupture [50]. (**iii**) Different states of the antenna: (**a**) free space, (**b**) bending radius R = 35 mm, and (**c**) attached to the right arm [52].

In the design of antennas, flexibility is the primary factor in the choice of substrate materials, which directly affects the performance of the wearable antenna such as efficiency, bandwidth, resistance to physical bending, and torsional effects. Among them, polyethylene terephthalate (PET) material itself has good properties such as transparency, firmness, and adaptability, and becomes the preferred antenna substrate, helping the antenna to achieve flexibility, wear-resistance, and reversible deformability [40]. Matyas’ team [41] (Figure 2i) developed a new method for how to mount nanomaterials into polymer substrates. They used solvothermal precipitation to synthesize 0-D silver nanoparticles for the first time. Particles ranging in diameter from 20 to 200 nm were then screened and printed onto PET using inkjet printing [42]. By this method, the fabricated antennas operate in the 2.02 GHz (−16.02 dB) and 2.3 GHz (−19.33 dB) frequency bands, and are not only soft but also as light as 0.208 g. Using the same substrate material and fabrication method, Guo et al. [43] designed an all-printed coplanar waveguide feed antenna suitable for Industrial Scientific Medical (ISM). Furthermore, to maximize the integration of wireless technology into smart applications for miniaturization, flexibility, light weight, and low cost, Jilani et al. [44] designed a millimeter-band flexible antenna for 5G wireless networks by exploiting the cost advantages of PET materials and utilizing inkjet printing technology. This antenna successfully combines multiple-input multiple-output and frequency reconfiguration schemes to achieve frequency selection and multi-channel transmission, providing a multifunctional and diverse network for wearable devices.

In addition to PET substrates, wearable antennas have also enabled the deployment of metal nanoparticles onto a variety of flexible materials, such as polydimethylsiloxane (PDMS), paper products, textiles, polymers, polyimides, and polyesters. PDMS has unique water resistance, heat resistance (up to 400 °C), ultraviolet (UV) resistance, and chemical stability due to its molecular structure, which makes PDMS still have considerable elasticity under extreme conditions. However, PDMS has the problem of poor adhesion to metals, so Simorangkir et al. [45] studied a method to embed conductive fabrics in PDMS. This design, which utilizes the conductive fabric as the heat sink and PDMS as the substrate, combined with a protective encapsulation, has good applicability to provide a robust flexible antenna or RF electronics for wearable applications. In addition, they combined PDMS and ceramics for antenna substrates, which reduced the size by more than 50% compared with pure PDMS and further demonstrated the versatility of the proposed technique. Although Simorangkir et al. solved the problem of poor metal adhesion, conductive polymer-based fabrics present a new problem of insufficient RF radiation due to the skin effect. Thus, Li et al. [46] designed a poly (3,4-ethylenedioxythiophene): polystyrene sulfonate (PEDOT:PSS) screen-printed fabric patch antenna. This antenna is based on high-frequency conductive fabric and uses a multi-strand structure and template to assist PEDOT:PSS phase separation, effectively improving the shortcomings of insufficient RF radiation and showing good flexibility and constant resonant frequency when simulating human body models. In addition, the Doppler radar system developed based on it shows satisfactory speed and distance detection accuracy. Compared with PDMS, paper substrates also have practical advantages in the design of portable devices. Specifically, paper-based materials have the characteristics of flexibility, thin structure, and low-cost, and are suitable for mass production with low carbon emissions. Baytöre et al. [47] used Mitsubishi photo paper with a thickness of 0.14 mm as the substrate of the wearable antenna, and combined inkjet printing technology to design a dual-frequency, coplanar, and flexible antenna. Furthermore, considering the good flexibility and robustness of polyester amide (PEA) material, Adhami et al. [48] fabricated a small coplanar waveguide-fed slot antenna using PEA instead of PET as the substrate. As a result, the antenna performs well in terms of gain, bandwidth, and operating efficiency in the bending case, and the size of the antenna is reduced to 20 × 10 mm^2^. It is worth mentioning that the antenna has a very low Specific Absorption Rate (SAR) value when it is close to the body.

In addition to considering the flexibility properties of the material, ductility is also an important factor because the antenna needs a certain level of cyclic strain to provide effective conductivity before it stretches and breaks [49]. Thus, Ramli et al. [50] (Figure 2ii) fabricated a microstrip patch antenna based on a silver ink–polysiloxane composite and a stretchable polysiloxane substrate, which has a conductivity of $\sigma =\sigma_{0}{\left(V-V_{c}\right)}^{S}$, where $\sigma_{0}$*,* $V_{c}$*,* and S are the conductivity of the sample, the conductivity of the filler, the percolation threshold, and the critical exponent, respectively. Because of the coupling agent and additives, the patch antenna produces very good adhesion between the ink and the substrate, preventing local breakage during stretching. Their experiments clearly show that the antenna can withstand mechanical deformation such as stretching, rolling, or twisting without breaking, and the frequency can be reconfigured, which is useful for applications such as wearable electronics, implantable medical devices, RF sensing, and interactive gaming. Zhou et al. [51] printed a flexible full-duplex antenna on knitted and non-woven fabrics using textile technology. Barium titanate (BT) dielectric ink, composed of acrylate, polyurethane (PU), and 4 wt.% 200 nm BT nanoparticles, is directly written and cured via UV light, greatly enhancing the dielectric properties of flexible fabric antennas. Moreover, the fabricated antenna can operate in both transmit and receive modes with very high isolation and is robust to bending, accompanied by a gain of 9.12 dB, which is superior to conventional textile antennas. The polymer nanocomposite substrate layer technique was exploited by Alqadami et al. [52] (Figure 2iii) in wearable flexible antenna arrays to achieve a maximum efficiency of about 60%, which is higher than similar textile-based antennas. This microstrip array topology with a full ground and intrinsically much larger bandwidth benefits from an innovative base layer design formed by a combination of iron oxide (Fe_3_O_4_) magnetic nanoparticles (25%) and PDMS (75%). In addition, Su et al. [53] fabricated a novel miniaturized wearable antenna (mosaic antenna) for cross-body communication on a polytetrafluoroethylene (PTFE) substrate for the first time using inkjet printing technology. Using machine learning techniques powered by Google TensorFlow, the antennas are designed to recognize human activity with 91.9% accuracy and are highly customizable, low-cost, and scalable.

Studies on the application of 0-D nanomaterials toward the development of wearable antennas in this section are tabulated in Table 1.

**Table 1:** Wearable antennas based on 0-D nanomaterials.

Nanomaterials	Antenna Base	Manufacturing Technology	Antenna Performance	Antenna Parameter	Application	References
AgNWs	PET	Inkjet printing	Soft and light	The antenna operates in two frequency bands, 2.02 GHz (−16.02 dB) and 2.3 GHz (−19.33 dB), with a weight of only 0.208 g.	Wearable electronic devices	[41]
			Flexibility, wearability, and reversible deformability	The reflection coefficient is −23 dB at 2.54 GHz, the −10 dB bandwidth is 530 MHz, and the obtained VSWR is 1.3.	Mobile communication	[43]
			Flexible, frequency reconfigurable	The antenna covers an overall bandwidth of 27.3–40 GHz in the four distinct modes with a measured peak gain of 6.2 dB at 34 GHz.	5G network	[44]
Conductive fabric	PDMS	Textile	Extremely bendable and machine washable	The antenna is small in size and more stable.	Wearable electronic devices	[45]
PEDOT:PSS	PET	Screen printing	Flexible, metal-free	The antenna shows a low RL of −50 dB and an estimated radiation efficiency of 28% at 2.35 GHz.	Wearable electronic devices	[46]
AgNWs	Mitsubishi Photo Paper	Inkjet printing	Soft, environmentally friendly, dual frequency	The antenna, made on a paper substrate with a relative permittivity of 3.6 and a loss tangent of 0.14, experiences a slight shift in resonant frequency when bent.	Wearable electronic devices	[47]
	PEA	Inkjet printing	Small size, low SAR value	The size of the antenna is 20 × 10 mm^2^, and it exhibits low SAR effects at 403 MHz and 2.45 GHz, measuring 0.25 W/kg and 0.33 W/kg, respectively.	Wireless biomedical devices	[48]
Polysiloxane–silver composite	PDMS	Inkjet printing	Stretch, roll, or twist	The resonant frequency of the antenna is 2.5 GHz, with an RL much lower than −15 dB. The bandwidth remains consistent with the unstretched condition when stretched at 10% and 20%.	Implantable medical devices	[50]
BT	-	Textile technology	High isolation	This flexible antenna can operate simultaneously in both transmit and receive modes, demonstrating robustness to bending.	Wearable device	[51]
Fe_3_O_4_	-	Polymer nanocomposite substrate layer technology	Wide bandwidth bottom SAR	The antenna measures 70 × 70 × 4.2 mm³, operates in the 5 GHz to 8.2 GHz frequency range, has a fractional bandwidth (FBW) of 50.34%, achieves a maximum radiation efficiency of 60%, and realizes a gain of 9.8 dB.	Telemedicine	[52]
AgNWs	PTFE	Inkjet printing	Low cost and scalability	The antenna achieves a precise human activity recognition accuracy of 91.9%.	Wearable device	[53]

### 3.2. 1-D Nanomaterial Structure Antenna

#### 3.2.1. Silver Nanowires

AgNWs have excellent properties in terms of printability, conductivity, light transmission, and resistance, and are considered to be the most promising materials in the field of printed electronics. Their surfaces are much smoother than those of etched copper foil antennas, resulting in less signal loss in high-frequency radios. Additionally, the signal loss of the AgNW antenna when heated at 100 °C is significantly lower than that of the silver paste antenna, which is composed of nanoparticles and flake. Against this background, AgNWs have become the most popular material for making wearable antennas. Qiu et al. [54] successfully designed, fabricated, and characterized meander-line dipole antennas using direct inkjet fabrication. The dual-band antenna based on graphene flakes and AgNWs operates in the 1.2 GHz to 1.5 GHz frequency band and 3.2 GHz to 3.8 GHz frequency band in wireless communication applications. Furthermore, the performance of meander wire antennas can be tuned by changing the mass ratio of CNTs and AgNWs in the aqueous dispersion mixture, which results in dipole antennas with great potential in radiolocation and 5G applications.

With the rapid development of wireless body area networks (WBANs) and wearable electronic devices, the demand for compact and high-performance flexible wearable antennas has increased substantially. To provide efficient in vitro communication, Jiang et al. [55] constructed a compact and flexible circularly polarized (CP) wearable antenna, which enabled the antenna to move relative to the human body during wearing. The improved robustness effectively solves the wireless link problem caused by the linear polarization that exists in all wearable antennas. This innovative antenna is fabricated from a low-loss composite material consisting of PDMS and AgNWs and has more stable performance and lower SAR than conventional CP patch antennas of the same size under bending and human loading.

**Figure 3:**
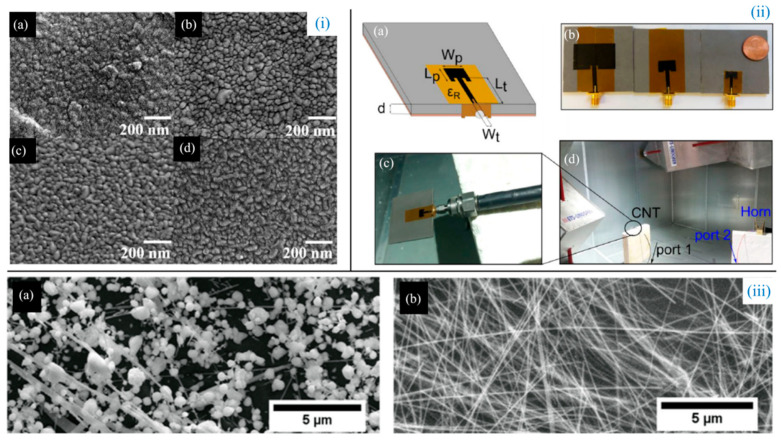
(**i**). SEM images of AZO at different substrate temperatures (**a**) 30 ℃, (**b**) 100 ℃, (**c**) 150 ℃, and (**d**) 200 ℃ [56]. (**ii**) Patch antenna design and testing configuration; (**a**) transmission line-fed patch antenna geometry and dimensions; (**b**) CNT patch antenna dimensions at each of the three target frequencies; (**c**) blow-up of the CNT antenna shown in (**d**); and (**d**) experimental set-up for radiation efficiency measurements inside the reverberation chamber [67]. (**iii**) (**a**,**b**) SEM images of AgNWs and composite film after annealing at 300 °C for 1 h [57].

In the traditional antenna manufacturing process, a large number of reagents are used on plastic substrates to etch copper films and form circuit patterns, causing serious environmental pollution and limiting their applications. Therefore, Wu et al. [56] (Figure 3i) prepared aluminum-doped zinc oxide (AZO)/AgNW composite transparent conductive films on glass substrates, thereby obtaining transparent, simple, pollution-free, and small-sized transparent wearable antennas. Multidimensional crystals and hybrid intercalators, such as AgNW/polyvinyl alcohol (PVA), TiO_2_/AgNW, and AgNW/ZnO, exist in the composite films, resulting in good optical transmittance and excellent mechanical flexibility of the antenna. On the other hand, the electromagnetic energy stored in the substrate, rather than the metallic path, can significantly reduce conduction losses. Coincidentally, in order to obtain better transparency and conductivity, Nair et al. [57] (Figure 3iii) formulated a nanocomposite printing ink of AgNWs and PEDOT:PSS. The sheet resistance of the printed film with 86% transparency is 23 Ω/sq, and the resistance change is less than 20% after 10,000 bending cycles, demonstrating good adhesion and mechanical deformation stability. The optimal transparency is calculated by the formula $\varphi =T^{10}/R_{S}$, where T is the transmittance (at 550 nm) and $R_{S}$ is the sheet resistance. Nanocomposites also exhibit better thermal stability, flatness, low contact resistance, and good optical transparency compared with pure AgNWs, and have been successfully applied to printed transparent flexible antennas radiating at Wi-Fi frequencies and printed transparent flexible heaters for anti-fog. Li et al. [58] used AgNWs and a slit structure to obtain higher transparency, and fabricated a broadband monopole antenna with a transparency of over 90%, making this antenna more widely used. With the good optical transmission and resistance properties of AgNWs, Dao et al. [59] fabricated an optically transparent patch antenna at millimeter wave frequencies. By characterizing the AgNW coating in the RF range up to 40 GHz, the insertion loss of the transmission line is measured. Simulation experiments show that the layer thickness of the single-layer AgNW-coated antenna is 0.04 μm, the electrical conductivity is 7 ms/m, and the light transmission rate is 67.9%. At the same time, physical experiments show that thinner coatings will reduce the efficiency of the antenna at 24 GHz, but it is acceptable above 60 GHz.

#### 3.2.2. Carbon Nanotubes

CNTs have attracted the attention of antenna engineers due to their light weight, low cost, corrosion resistance, good temperature stability, tunable electrical conductivity, extraordinary flexibility and elasticity, low thermal expansion coefficient, and high tensile strength [60]. In earlier studies, the performance of CNT antennas was not comparable to that of metals like copper. However, recent improvements in the processing of liquid-phase CNTs have led to the production of macroscopic CNT materials with enhanced orientation and electrical conductivity. To further highlight CNT advantages, Mansor et al. [61] fabricated wearable monopole antennas with CNTs and copper as conductive materials, respectively. The experimental results show that the two antennas have good consistency in characteristics. However, when the ground structure was improved by introducing horizontal slit cuts and vertical stubs, the bandwidth of the wearable antenna fabricated with CNTs was significantly improved. Meanwhile, when the antenna is applied to the upper arm region of the voxel manikin, the reflection coefficient characteristic drops by about −10 dB. With the continuous development of CNT manufacturing technology, inkjet printing technology has gradually become popular [62]. Gong et al. [63] made a dual-frequency conformal antenna by inkjet printing CNTs on a flexible substrate, and the experimental results showed that the radius of the conformal cylindrical surface and the conductivity of CNTs have little effect on the antenna performance. With further integration on textile substrates, the designed antenna is even more superior to traditional metal-fabricated antennas in terms of its lightweight, low-cost, and conformal properties.

It has been demonstrated in previous studies that copper dipole antennas do not provide reliable efficiency when transmitting or detecting terahertz frequency signals due to the skin effect. Therefore, the single-walled carbon nanotube (SWCNT) dipole antenna was built to meet the small size and lightweight requirements without skin effect, but the actual efficiency did not reach the expected goal. On the other hand, the radiation efficiency of SWCNT antennas is very low, which is mainly attributed to the radiation resistance reduced by the strong retardation of surface waves. However, if a single CNT is used to build an antenna, the problem of impedance mismatch occurs because its characteristic impedance (10–100 kΩ) is very large compared with that of standard transmission lines [64]. In this context, the idea of making dipole antennas work with high frequency and high efficiency by coating SWCNT came up [65]. Hajjyahya et al. [66] compared the efficiency versus frequency of SWCNT-coated and uncoated dipole antennas of different lengths and constant radii and showed that the coating enables the efficiency of the antenna to be about 59% with zero loss and about 77% with impedance mismatch. This higher efficiency value and faster data rate will be very effective for biomedical engineering.

Although the skin effect cannot be completely eliminated, Bengio et al. [67] (Figure 3ii) fabricated a microstrip patch antenna using CNT films and performed radiation efficiency measurements to test the reduction in the skin effect. Their experimental results showed a measured radiation efficiency of 94% at 10 GHz and 14 GHz, matching the equivalent copper antenna. In addition, the minimum CNT film thickness required to match the properties of copper increases with frequency due to reduced losses from the skin effect.

Wearable forms, application methods, and application environments all place special requirements on wearable devices. As one of the key components in wearable devices, antennas are also required to withstand harsh environments. Chowdhury et al. [68] applied circularly polarized antennas to wireless communication systems and constructed an ITO E-shaped nanoantenna based on CNTs. The antenna uses the CNT layer as the cover layer of the Indium Tin Oxides (ITOs) patch to improve the conductivity and realize circular polarization by loading E-type grooves and I-type grooves in the ITO patch and the CNT layer. The experimental results show that the gain and bandwidth are significantly improved, and the cross-polarization suppression greater than 30 dB is also obtained, which confirms that the antenna has good circular polarization performance. This also further improves the ability to suppress multipath interference and ensure stability in harsh weather conditions, making this new antenna more suitable for terahertz applications. In addition, Aïssa et al. [69] fabricated a flexible antenna by using PDMS and liquid metal. The antenna is fabricated by directly writing liquid metal alloy (EGaIn)/SWCNT nanomaterial composites into a PDMS substrate with the assistance of ultraviolet rays. When dealing with harsh environments, it can better withstand severe mechanical shock by bending rather than breaking. The experimental results show that the composite conductivity and reflection coefficient increase with the increase in SWNT concentration. In addition, single-walled CNTs have a long-term effect on the stability of the mechanical properties of the prepared samples. Not exactly the same, Olenick et al. [70] designed the antenna with non-metal as the conductive composite material. Monopole and dual-band F-shaped antennas were fabricated on 2 mm thick polypropylene substrates, using thermoplastic PU as a matrix and multi-walled CNTs as conductive fillers, but the antenna performance was poor in harsh environments. In terms of fabrication technology, Bayram et al. [71] proposed a conformal and lightweight flexible antenna technology based on electronic fabric conductors and composites. Electronic fabric conductors are made of SWNT and silver-coated fabrics, which ensure good electrical conductivity while satisfying mechanical and structural flexibility. At the same time, good structural integrity and excellent RF performance provide the antenna with strength in harsh environments.

As the communication end of wearable devices, the potential interference and safety problems caused by the retractable antenna emitting radio near the human body are particularly important for materials that can shield electromagnetic interference. Therefore, Li et al. [72] prepared a stretchable material for shielding electromagnetic interference based on CNTs for wearable antennas. The material is formed by depositing Ti_3_C_2_Tx nanosheets (MXene) and SWNT onto latex to form a wrinkled textured coating. The coating exhibits good mechanical flexibility while shielding electromagnetic interference. Also, in order to eliminate signal interference, Tanaka et al. [73] proposed an adaptive control scheme for the angular sensitivity of nanoantennas based on CNTs. The scheme describes the characteristics of the antenna using the single-clip metal sphere model and the image-charge method. The experimental results show that the most sensitive directions are between −π/2 and π/2, and the shape of the sensitivity can be controlled to be omnidirectional or correspond to a Hertzian dipole antenna. This research promises to help develop nanoscale adaptive antennas to cancel out interfering signals.

Studies on the application of 1-D nanomaterials toward the development of wearable antennas in this section are tabulated in Table 2.

**Table 2:** Wearable antennas based on 1-D nanomaterials.

Nanomaterials	Antenna Base	Manufacturing Technology	Antenna Performance	Antenna Parameter	Application	References
AgNWs	PDMS	Inkjet printing	Bendable, high radiation efficiency	The antenna can operate in the frequency ranges of 1.2 GHz to 1.5 GHz and 3.2 GHz to 3.8 GHz, with S11 > 10 dB.	Wireless location and 5G	[54]
		Mold	Low SAR value, stable performance	The antenna achieves S11 < −15 dB, axial ratio less than 3 dB, and a gain of approximately 5.2 dB.	Medical	[55]
AgNW/PVA	Glass baseboard	Magnetron sputter deposition	Transparent, simple, pollution-free, and small	The AZO/AgNW stacked film has a resistivity of 2.15 × 10^−4^ Ω·cm and a transmittance of 80.28% in the range of 400 to 800 nm. The transparent antenna constructed with this AZO/AgNW stacked film operates at a frequency of 2.4 GHz.	Glass coating, mobile phone, electronic label	[56]
Ag-NW and PEDOT:PSS	-	Inkjet printing	High transparency and high conductivity	This conductive film has excellent adhesion and outstanding mechanical deformation stability, with a resistance change of less than 20% after 10,000 bends.	Flexible optoelectronic devices	[57]
AgNWs	-	Inkjet printing	Ultra-wide bandwidth, flexibility, and transparency	This antenna has a bandwidth of up to 26 GHz (18 GHz to 44 GHz), a high radiation efficiency of 55%, a maximum gain of 1.45 dB, and transparency of over 90%.	Windows, solar cells	[58]
		Spin coating process	Low resistance value, high antenna efficiency	The radiation efficiency of the antenna is 8.9% at 24 GHz and 49.4% at 61 GHz.	Photovoltaics, displays, and touchscreens	[59]
CNT	PDMS	CPW feed structure excitation	Bandwidth improvement	The antenna bandwidth has increased by 18%.	Body wireless applications	[61]
	Flexible paper substrate	Inkjet printing	Lightweight, low cost, and conformal properties	The curvature radius of the conformal cylindrical surface and the conductivity of CNTs have a relatively minor impact on the antenna performance in the 2.45 GHz and 5.8 GHz frequency bands.	Wearable electronic devices	[63]
	-	Coating	High efficiency and faster data transfer rate	The efficiency of the coated CNT dipole antenna is approximately 59% under perfect matching conditions and around 77% under unmatched impedance conditions with zero loss.	Biomedical engineering	[66]
		-	High radiation efficiency	The antenna has a radiation efficiency of 94% in the 10 GHz and 14 GHz frequency bands.	Aerospace	[67]
		Coating	Significantly improved gain and bandwidth	The antenna has a 10 dB impedance bandwidth of 22.2% and a 3 dB axial ratio bandwidth of 9.14%.	Terahertz applications	[68]
SWNT	PDMS	Direct write Technology	Strong flexibility	The antenna operates at a frequency of 4 GHz.	Implantable medical devices	[69]
MWCNT	PU	Dip coating technology	Small in size but less flexible	The monopole antenna has measured gains of −10.0 dB and −5.5 dB in the 2.45 GHz and 5.18 GHz frequency bands, respectively.	Wearable electronic devices	[70]
SWNT	-	Textile technology	Strong conductivity and flexibility	The patch antenna has a gain of 6 dB.	Automated aerial vehicle	[71]
MXene/SWNT	Latex	Deposition technology	Can shield electromagnetic interference	The S-MXene antenna was highly stretchable (up to 150% uniaxial strain) and demonstrated strain-independent independent reflected power of less than 0.1% as well as remained stable during fatigue tests.	Wearable electronic devices	[72]

### 3.3. 2-D Nanomaterial Structure Antenna

New wireless communication systems based on body area networks are appearing more and more frequently in our daily lives to meet the growing demands of smart telemedicine and health monitoring, in which wearable devices play an important role [74]. In wireless bulk networks, devices need to meet some special properties such as abrasion resistance, flexibility, low profile, and low SAR. Therefore, a lot of research has been conducted on wearable devices, especially antenna design [75]. Wearable antennas based on flexible substrates have attracted much attention due to their flexibility when the soft substrate is coated with metal films. However, with the increased performance requirements of lightweight, bending resistance, and corrosion resistance properties, traditional metal materials are no longer suitable. In recent years, new conductive materials have been gradually developed, such as metal nanoparticles, conductive polymers, CNTs, and graphene. Among them, metal nanoparticles are too expensive and easily oxidized [76], conductive polymers generally have low electrical conductivity [77], and CNTs have relatively high sheet resistance caused by junction resistance [78], all of which are not suitable for antenna design. Graphene is a newly discovered single-layer, two-dimensional nanomaterial with excellent mechanical and optical properties. Compared with traditional antenna materials like copper and aluminum [79], wearable antennas made from graphene offer multiple advantages. Firstly, the electromagnetic characteristics can enhance the millimeter wave and sub-millimeter wave bands in 5G communication networks. Additionally, graphene nanomaterials are more flexible and can be easily integrated into clothing and other flexible media. Lastly, the elongation of graphene nanomaterials can reach up to 120%, with conductivity a million times higher than that of copper and strength nearly 100 times larger than that of steel. Therefore, graphene has become the optimal material for wearable antennas [80].

In recent years, terahertz (THz) technology has attracted high research interest due to its increasing applications in spectroscopy, medicine, earth and space science, defense, communications, material characterization, sensing, and imaging [81]. Terahertz signals have non-ionizing properties, high penetrability and low attenuation, and high-resolution imaging capabilities. For antennas in this range, materials currently used include copper, graphene, and CNTs. For copper metal, the reduced skin depth and electrical conductivity lead to large propagation loss and reduced radiation efficiency of the antenna at terahertz frequencies. These facts drive the use of carbon materials in terahertz antennas, such as CNTs and graphene [82]. At terahertz frequencies, CNTs have higher electrical conductivity and dynamic inductance; therefore, the fabricated antenna outperforms copper antennas [83]. Graphene, the latest reported material for terahertz antenna design, supports very slow plasmonic waves and is expected to perform better than copper and CNTs for making terahertz antennas. Thus, Dash et al. [84] compared a THz antenna composed of copper, graphene, and CNTs with a typical dipole antenna with a resonant frequency of 1 MHz, and the experimental results showed that graphene obtained the optimal directionality and miniaturization. In order to clearly compare the performance of antennas made of graphene materials and copper materials, Atser et al. [85] used the high-frequency structure simulator HFSS to study the RL, bandwidth, gain, directivity, and VSWR of the two antennas. The experimental results show that the classical copper antenna resonates at lower frequencies with an RL of −14.7028 dB and a corresponding VSWR of 3.2336, achieving a gain of 4.6219 dB in a bandwidth of 1188.5 GHz. While the graphene-based patch antenna resonates at higher frequencies, the RL is −24.4555 dB, the corresponding standing wave ratio is 1.0413, and the maximum gain is 7.1943 dB at 522.3 GHz bandwidth. It can be seen from the data that graphene is a better choice for replacing copper materials for patch antennas in wireless applications in the terahertz band. Abohmra et al. [86] designed a flexible antenna based on graphene and explored possible resonant frequencies in the terahertz band by changing the chemical potential and relaxation time of graphene. The experimental results show that the antenna can resonate at three different frequencies: 4.546 THz, 4.636 THz, and 5.347 THz. In addition, the antenna bandwidth is 20 GHz, and the radiation efficiencies in free space and the human body are 96% and 50%, respectively, with corresponding gains of 7.8 dB and 7 dB. In the overall evaluation, the antenna is suitable for short-distance wireless communication near the human body due to its compact and flexible structure, good impedance matching, high bandwidth and gain, and good efficiency. In addition, the team evaluated the effect of the substrate material on the antenna performance and found that the antenna based on the polyamide substrate had a maximum reflection coefficient of 42 dB.

**Figure 4:**
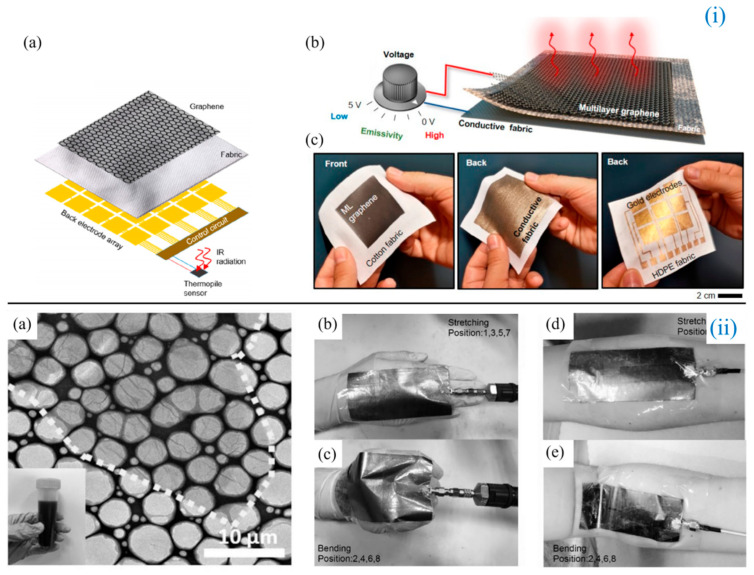
(**i**) (**a**) Illustration of a textile device with sensing and display capabilities; (**b**) illustration of the textile device with various laminated layers: multilayer graphene, fabric separator, and back electrode layer; (**c**) representative examples of fabricated devices on various textile materials such as woven cotton fabric and nonwoven high-density polyethylene fabric. Continuous conductive textiles or patterned gold electrodes can be used as the back electrode [93]. (**ii**) (**a**) Transmission electron microscope image of graphene oxide; photographs of an FGF antenna sensor attached to (**b**,**c**) the back of the hand joint based on paper; and (**d**,**e**) the inside of the elbow based on PET [87].

Although graphene has excellent electronic, mechanical, and optical properties, when graphene is used in commercial electronic devices, single-layer or multi-layer graphene films exhibit high sheet resistance and insufficient electrical conductivity, which greatly limits the applications in wearable antennas. Therefore, many researchers have tried various methods to solve the problem of the low electrical conductivity of graphene. However, the electrical conductivity of these graphene films is still much lower than that of metal materials (107 S/m). Tang et al. [87] (Figure 4ii) fabricated flexible multilayer graphene films (FGFs) using high-temperature heat treatment and a subsequent rolling process of graphene oxide assembled films and tried to lay FGFs on paper substrates to fabricate rectangular microstrip patch antenna sensors. The physical length L and width W of the antenna are designed as $L=\frac{c}{2f_{res}\sqrt{\varepsilon_{reff}}}-2∆L$, $W=\frac{c\sqrt{2}}{2f_{res}\sqrt{\varepsilon_{r}+1}}$, $\varepsilon_{r}$ is the dielectric constant of the cellulose paper, $\varepsilon_{reff}$ is the effective dielectric constant, c is the speed of light in free space, and ∆L is the extended length. The sensor outperforms the copper antenna sensor in compression and tension, truly achieving high conductivity and good stretchability. Furthermore, FGF antennas exhibit mechanical flexibility, reversible deformability, and structural stability, making them ideal for applications such as wearable devices and wireless strain sensing. Fang et al. [88] designed a compact and thin ultra-wideband (UWB) antenna with a size of 32 × 52 × 0.28 mm^3^ based on graphene-assembled film (GAF) and tested it for wearable applications. The team introduced two H-shaped slots on the coplanar waveguide feed structure to tune the current distribution and thus increase the antenna bandwidth. The UWB properties were further confirmed by in vivo measurements, showing a bend-insensitive bandwidth of about 67%. In the flat and curved states at 7.45 GHz, the maximum gain is 3.9 dB and 4.1 dB, respectively. The experimental results show that the antenna can work normally close to the human body and can withstand repeated bending, which is very suitable for application in wearable devices. In contrast, the GAF antenna designed by Wang et al. [89] has better RL and radiation capability in the 5G communication band and ISM band. The size of the antenna is only 50.5 × 48.5 × 2.08 mm^3^. In addition, due to the artificial magnetic conductor (AMC) structure, small-lobe radiation suppression and main-lobe radiation enhancement are achieved. The experimental results show that the GAF wearable antenna has a low SAR value that conforms to the international IEEE standard, and has good radiation ability under bending state and human body load. Zhang et al. [90] designed a similarly small size (50 × 50 mm^2^) flexible GAF antenna for use in wireless wearable sensors with a tensile bending strain sensitivity of 34.9 and compressive bending strain sensitivity of 35.6. The sensor was attached to the back of the hand and the wrist and showed good mechanical flexibility and structural stability after multiple bending tests. Li et al. [91] also reported a flexible NFC tag antenna based on highly conductive graphene-assembled films (HCGAF). Their prototype fabricated by a one-step laser direct die engraving method showed a 10 dB bandwidth centered at 13.70 MHz at 2.5 MHz and a quality factor (Q) of 9.19. Experimental results show that the maximum read range of HCGAF NFC tags is about 7.5 cm, which is comparable to commercial metal NFC tags. In addition, the team further demonstrated the practical application of a key card and electronic business card close to the human body.

In wearable antenna designs, single-layer antennas, such as monopoles and dipoles, usually do not have a back metal to isolate the antenna from the human body, resulting in high SAR. AMC-based wearable antennas can solve this problem, but this multi-layer structure increases the overall thickness, which cannot guarantee the performance of the antenna in motion once the thickness exceeds 4 mm and the linear polar works in a chemical manner. Thus, Zu et al. [92] proposed a flexible, low SAR, low profile, and circularly polarized wearable antenna. The selected materials are highly conductive graphene films and PDMS substrates, which possess good flexibility and mechanical stability and are lightweight. The measurement results show that the SAR peaks are 1.02 W/kg and 0.947 W/kg, respectively, when the load is 1 g and 10 g on the human body, which is in line with the international SAR. Also, to reduce SAR, Ergoktas et al. [93] (Figure 4i) proposed a unique lamination method using ML graphene to fabricate infrared textile devices, including displays, yarns, and stretchable devices. This textile attachment technique opens up new manufacturing possibilities. Ibanez-Labiano et al. [94] introduced CVD-grown ML graphene sheets on a cellulose textile substrate for the first time, creating a soft, fabric-based communication interface that does not compromise tactile comfort. The antennas cover the frequency band from 3 GHz to 9 GHz, providing an efficient solution for future high data rates and efficient communication links. In addition, the overall effect of the antenna is small when it is bent and close to the human body, so it is inferred that the scheme of the textile communication interface based on the flexible graphene antenna is feasible. In biotelemetry applications, Bala et al. [95] designed a circularly polarized microstrip line-fed curved patch based on graphene with a small size, high directivity, small SAR, and low power consumption. The antenna has good impedance matching, reasonable gain, and high radiation efficiency in the GHz broadband frequency range with a reduced SAR, making it suitable for ultra-wideband (UWB) health monitoring systems.

With the widespread interest in wearable electronics, there is a growing demand for cost-effective textile antennas that can withstand stretching, moisture, and bending [96]. Akbari et al. [97] conducted the first study of graphene-based passive ultra-high frequency radio frequency identification (RFID) tags on fabric substrates. The team deposited conductive inks containing functionalized graphene nanosheets directly on cotton fabric substrates to fabricate tag antennas. Label performance was then evaluated by measuring changes before and after high humidity, bending, and stretching. The experimental results show that the wireless tag is not suitable for stretching, but this low-cost and environmentally friendly graphene RFID tag has a remarkable and unique response to moisture and high reliability under severe bending conditions. Kapetanakis et al. [98] designed textile rectangular, triangular, and circular probe-fed patch antennas. A comparative study of graphene-textile, all-textile, and copper-textile antennas found that the graphene sheet-like patch prototype was corrosion-resistant and the circular patch had very good performance under bending conditions. In addition, the circular graphene patch felt substrate CGSF1 prototype achieves a measurement bandwidth of 109 MHz, a gain of 5.45 dB, an efficiency of 56%, and excellent bending performance. Német et al. [99] also designed 5G wireless band antennas based on textiles. The antenna uses denim and graphene as the basic materials, which satisfies flexibility and easy integration into clothing. On the other hand, graphene is environmentally friendly and biodegradable due to its non-metallic properties and is affordable in terms of manufacturing costs. Structurally, the antenna consists of a coplanar waveguide (CPW) feed patch with truncated edges and a pair of L-shaped stubs. As a result, the antenna has a bandwidth of 3.3–3.8 GHz, a peak gain of 3.17 dB at 3.7 GHz, and an efficiency of 64%. Labiano et al. [100] constructed an ultra-wideband antenna using graphene as a conductive patch. The antenna uses cotton fabric as the base material to meet the flexibility requirements, with a bandwidth of 2–8 GHz and an efficiency of about 60%.

Printed flexible graphene antennas for communication systems have become the focus of science and technology due to their good electrical properties, but more importantly, their excellent mechanical properties. Recently, graphene-based RFID antennas [101,102], RF transmission lines and antennas [103], and fully integrated RFID devices have been demonstrated. The conductivity of printed graphene flakes is significantly lower than that of copper, aluminum, or even printed metal inks, which are by far the most commonly used conductor materials in flexible antennas. The raw material cost of graphene ink is very low, in contrast, the cost of silver ink is relatively high, which depends largely on the price level of bulk silver metal. In addition, graphene-based structures have advantages such as chemical stability, mechanical flexibility, and fatigue resistance. Therefore, Lamminen et al. [104] designed a screen-printed broadband elliptical dipole antenna based on graphene flakes. In this technology, the CPW is printed on the Kapton substrate with a graphene slurry screen, and then heat-treated and rolled to form a graphene flake structure with a resistivity and thickness of 4 Ω·m and 10 μm, respectively. The printed antenna has a maximum measured gain of 2.3 dB at 4.8 GHz with an efficiency of 60%. Furthermore, the total RF link loss of the graphene sheet-printed antenna is only 3.1 dB compared with the commercial silver ink screen-printed antenna. This indicates that the electrical properties of the screen-printed graphene flakes will not degrade after repeated bending, which is suitable for realizing low-cost wearable RF wireless communication devices. Overall, graphene ink formulations are one of the most convenient and promising approaches to manufacturing devices. On the other hand, the production method of high-quality graphene inks is crucial for printing and coating freestanding conductive graphene films [105]. Tung et al. [106] improved the graphene ink formulation and reported a flexible and efficient broadband slot antenna based on highly conductive composites composed of PEDOT and N-doped hetero-reduced graphene oxide (N-doped rGO). The hybrid material exhibits high electrical conductivity, a low sheet resistance of 0.56 Ω/square, a thickness of 55 μm, and good mechanical elasticity (resistance change <5.5% after 1000 bending cycles), confirming that the composite is a suitable antenna conductor. The antenna achieves an estimated conduction efficiency of nearly 80% over a bandwidth of 3 to 8 GHz. Furthermore, N-doped rGO sheets enhanced mechanical stability, while PEDOT acted as an additive and/or binder to improve electrical and mechanical properties compared with graphene and PEDOT. This high-performance nanocomposite meets the requirements for antenna design and has successfully operated in free space as part of a wearable camera system.

Graphene nanomaterials, characterized by their ultra-lightweight, high electrical conductivity, and flexibility properties, have broadened the applications of graphene antennas. Specifically, wearable antennas can adapt to various frequency bands and are perfectly compatible with current wireless communication standards such as 5G and Wi-Fi [107,108]. For IoT applications, these antennas not only provide stable wireless connections but also contribute to the miniaturization of the overall devices [109]. Additionally, graphene antennas offer greater data throughput and a wider connection range, expanding possibilities for wearable technology, smart cities, and autonomous vehicles [110,111]. Despite these advantages, the biocompatibility and safety still need to be strengthened. Future researchers could explore modifying the size, shape, and surface properties of graphene to enhance biocompatibility. On the other hand, biocompatible materials could be introduced as a medium for contact with human skin, ensuring safety. Regular safety assessments and monitoring are crucial to identify and address potential health issues promptly. For users, the usage time of these antennas should be limited, and especially, they should be worn away from sensitive areas like the head or heart. Finally, understanding the correct usage methods and raising awareness about health and safety concerns are also critically important.

Studies on the application of 2-D nanomaterials toward the development of wearable antennas in this section are tabulated in Table 3.

**Table 3:** Wearable antennas based on 2-D nanomaterials.

Nanomaterials	Antenna Base	Manufacturing Technology	Antenna Performance	Antenna Parameter	Application	References
Graphene	-	-	Directionality and miniaturization	The resonant frequency of the antenna is 1 terahertz.	Defense, communications	[84]
	Silicon dioxide	-	Higher gain than copper patch	The antenna achieves a maximum RL of −24.4555 dB with the corresponding VSWR of 1.0413. The maximum gain of 7.1943 dB is achieved with a bandwidth of 522.3 GHz.	Wearable electronic devices	[85]
	Polyesteramide	-	Good impedance matching, high bandwidth, and gain	The proposed antenna proved the tunability of the graphene antenna to resonate at different frequencies in the terahertz band, 4.546 THz, 4.636 THz, and 5.347 THz, by varying the chemical potential and relaxation time.	Short-range wireless communication	[86]
	Graphene film	Rolling process	High conductivity and good stretchability	The antenna operates at a frequency of 1.63 GHz, with strain sensitivities of 9.8 and 9.36 during bending and stretching, respectively.	Wearables and wireless strain sensing	[87]
GAF	Graphene film	-	Small size and good bending properties	The antenna can work properly within the ultra-wide range from 4.0–8.0 GHz with a maximum measured gain of 4.1 dB.	Wearable device	[88]
			Good RL and radiation capability	The antenna has dimensions of 50.5 × 48.5 × 2.08 mm³, and it exhibits good RL and radiation capability in the 5G communication band (3.5 GHz) and the ISM band (5.8 GHz).	5G	[89]
GAF	-	-	Small size and high sensitivity	The antenna operates in the frequency range of 3.13–4.42 GHz, with dimensions of 50 × 50 mm^2^. The strain sensitivity during tensile bending and compressive bending is 34.9 and 35.6, respectively.	Wearable electronics and the Internet of Things	[90]
HCGAF	PET	Laser engraving	Wireless Body Center Network	The antenna has a 10 dB bandwidth of 2.5 MHz, a resonant frequency of 13.70 MHz, and a quality factor of 9.19.	Identification	[91]
Graphene	PDMS	-	Good flexibility, mechanical stability, and lightweight	The antenna has dimensions of 2.57 mm. Within the range of 5.75 to 5.83 GHz, the axial ratio is less than 3 dB, the reflection coefficient is less than −15 dB, and it achieves a gain range of 5.0–6.1 dB.	Human body’s communication system	[92]
	-	Chemical vapor deposition	High data rates and efficient communication	The antenna covers a bandwidth from 3–9 GHz.	Health monitoring	[94]
		CST	Small size, high directivity, small SAR	The antenna exhibits an RL of −25.05 dB at the resonant frequency of 2.4 GHz and −25.17 dB at the second resonant frequency of 3.94 GHz.	Biotelemetry	[95]
	Fabric base	Deposition method	Not suitable for stretching but bendable	The antenna is cost-effective and environmentally friendly.	Wearable sensor	[97]
		Textile technology	Lightweight and mechanical stability	The antenna has a diameter of 55.3 mm, a bandwidth of 109 MHz, a gain of 5.45 dB, an efficiency of 56%, and covers the entire ISM band in a bent state, with a SAR of less than 0.003 W/Kg.	Medical	[98]
		Textile technology	Flexible and easy to integrate	The antenna has a bandwidth of 3.3–3.8 GHz, a peak gain of 3.17 dB at 3.7 GHz, and an efficiency of 64%.	5G wearables	[99]
		Textile technology	Soft and high transfer efficiency	The antenna has a bandwidth of 2–8 GHz.	Biomedical Science	[100]
	Kapton substrate	Screen printing	Highly conductive antenna with high efficiency	The measured maximum antenna gain is 2.3 dB at 4.8 GHz.	Wearable communication devices	[104]
PEDOT/Graphene	Teflon substrate	-	High flexibility and robustness	The antenna achieves close to 80% efficiency in the bandwidth range of 3.8–6.2 GHz.	Wearable device	[106]

## 4. Conclusions

With the growing research in wearable and implantable devices, there is a significant demand for wearable antennas with high performance, low SAR, enhanced comfort, miniaturization, and integration with the human body. However, wearable antennas face several challenges. Firstly, antenna miniaturization necessitates higher integration in a smaller area, posing stringent requirements on antenna design and fabrication. Secondly, the performance of wearable antennas is significantly affected by the human body, necessitating the consideration of antenna–body interactions to ensure stable and reliable antenna operation. Moreover, wearable antennas need to meet the demands of multi-band and multi-mode communication, increasing the complexity of antenna design. Additionally, the safety and comfort of wearable antennas are of utmost importance, as skin allergies, discomfort during wearing, and electromagnetic radiation are potential concerns. Therefore, comfort factors must be carefully considered to ensure comfortable and safe antenna usage. Lastly, wearable antennas should exhibit environmental friendliness and sustainability to align with green development goals.

The above difficulties are gradually being solved by the application of nanomaterials. Firstly, nanomaterials possess superior electrical properties, enabling higher performance in compact sizes, which aids in the miniaturization of wearable antennas. Secondly, the flexibility and conformability of nanomaterials make the antennas more suitable for matching the human body’s contours, enhancing wearable comfort. Furthermore, nanomaterials offer strong integration capabilities, facilitating the realization of integrated antenna designs and addressing the challenges of space constraints and integration with other components. Extensive research has shown that adding nanomaterials to antennas significantly improves antenna parameters. Commonly used nanomaterials include metal nanoparticles, CNTS, AgNWs, and graphene. Wearable antennas based on metal nanoparticles are more readily integrated with flexible substrates than conventional antennas, resulting in smaller and more compact structures. They also offer advantages in terms of fabrication complexity and cost. Antennas fabricated using AgNWs exhibit excellent transparency, conductivity, and printability, enabling reduced signal loss while achieving miniaturization. Owing to the exceptional strength, high electrical conductivity, and lightweight nature of CNTs, antennas based on CNTs exhibit reduced power consumption and enhanced radiation efficiency. Additionally, fabricated patch antennas demonstrate improved impedance bandwidth without compromising radiation characteristics. Given the outstanding mechanical flexibility, conductivity, transparency, strength, and tunable electronic properties of graphene nanomaterials, they are a prime choice for constructing flexible systems. Antennas based on flexible multilayer graphene films exhibit higher strain sensitivity to compression and tensile bending, enabling reduced antenna losses while improving wideband impedance matching.

In conclusion, nanomaterials, with their superior electronic and optical properties compared with conventional materials, are highly suitable for flexible wearable antennas. The technology of flexible wearable antennas based on nanomaterials holds great promise for future applications in human healthcare, motion detection, human–machine interaction, and various other fields.

## Data Availability

All data required to determine the conclusions in the paper are presented in the paper.

## References

[1] Wang C., Ma B., Zhai R., Li R., Zhang N., Zhang K., Zhang S. (2023). Experimental approach of contact mechanics for polyethylene materials with human skin under wet condition. J. Adhes. Sci. Technol..

[2] Wang C., Liu C., Shang F., Niu S., Ke L., Zhang N., Ma B., Li R., Sun X., Zhang S. (2023). Tactile sensing technology in bionic skin: A review. Biosens. Bioelectron..

[3] Zhang S., Liu C., Sun X., Huang W. (2022). Current Development of Materials science and engineering towards epidermal sensors. Prog. Mater. Sci..

[4] Hasni U., Topsakal E. Wearable antennas for on-body motion detection. Proceedings of the 2020 IEEE USNC-CNC-URSI North American Radio Science Meeting (Joint with AP-S Symposium).

[5] Ashyap A.Y., Zainal Abidin Z., Dahlan S.H., Majid H.A., Saleh G. (2019). Metamaterial inspired fabric antenna for wearable applications. Int. J. RF Microw. Comput.-Aided Eng..

[6] Saeidi T., Al-Gburi A.J.A., Karamzadeh S. (2023). A Miniaturized Full-Ground Dual-Band MIMO Spiral Button Wearable Antenna for 5G and Sub-6 GHz Communications. Sensors.

[7] Mahmood S.N., Ishak A.J., Saeidi T., Alsariera H., Alani S., Ismail A., Soh A.C. (2020). Recent advances in wearable antenna technologies: A review. Prog. Electromagn. Res. B.

[8] Li W., Zu H., Liu J., Wu B. (2021). A low-profile ultrawideband Antenna based on flexible graphite films for on-body wearable applications. Materials.

[9] Al-Dulaimi Z., Elwi T.A., Atilla D.C. (2022). Design of a meander line monopole antenna array based hilbert-shaped reject band structure for MIMO applications. IETE J. Res..

[10] Ali S.M., Sovuthy C., Imran M.A., Socheatra S., Abbasi Q.H., Abidin Z.Z. (2020). Recent advances of wearable antennas in materials, fabrication methods, designs, and their applications: State-of-the-art. Micromachines.

[11] Yan S., Soh P.J., Vandenbosch G.A. (2018). Wearable ultrawideband technology—A review of ultrawideband antennas, propagation channels, and applications in wireless body area networks. IEEE Access.

[12] El Gharbi M., Fernández-García R., Ahyoud S., Gil I. (2020). A review of flexible wearable antenna sensors: Design, fabrication methods, and applications. Materials.

[13] Yang M., Ye Z., Ren Y., Farhat M., Chen P.-Y. (2023). Recent Advances in Nanomaterials Used for Wearable Electronics. Micromachines.

[14] Cao X., Halder A., Tang Y., Hou C., Wang H., Duus J.Ø., Chi Q. (2018). Engineering two-dimensional layered nanomaterials for wearable biomedical sensors and power devices. Mater. Chem. Front..

[15] Zhang S., Xia Q., Ma S., Yang W., Wang Q., Yang C., Jin B., Liu C. (2021). Current advances and challenges in nanosheet-based wearable power supply devices. Iscience.

[16] Zhang S., Zhao W., Zeng J., He Z., Wang X., Zhu Z., Hu R., Liu C., Wang Q. (2023). Wearable non-invasive glucose sensors based on metallic nanomaterials. Mater. Today Bio.

[17] Garg A., Chalak H., Belarbi M., Zenkour A., Sahoo R. (2021). Estimation of carbon nanotubes and their applications as reinforcing composite materials–an engineering review. Compos. Struct..

[18] Fahad S., Yu H., Wang L., Haroon M., Ullah R.S., Nazir A., Naveed K.-u.-R., Elshaarani T., Khan A. (2019). Recent progress in the synthesis of silver nanowires and their role as conducting materials. J. Mater. Sci..

[19] Asiya S., Kyzas G.Z., Pal K., de Souza F.G. (2021). Graphene functionalized hybrid nanomaterials for industrial-scale applications: A systematic review. J. Mol. Struct..

[20] Azadmanjiri J., Reddy T.N., Khezri B., Děkanovský L., Parameswaran A.K., Pal B., Ashtiani S., Wei S., Sofer Z. (2022). Prospective advances in MXene inks: Screen printable sediments for flexible micro-supercapacitor applications. J. Mater. Chem. A..

[21] Prunet G., Pawula F., Fleury G., Cloutet E., Robinson A.J., Hadziioannou G., Pakdel A. (2021). A review on conductive polymers and their hybrids for flexible and wearable thermoelectric applications. Mater. Today Phys..

[22] Nonappa (2023). Precision nanoengineering for functional self-assemblies across length scales. Chem. Commun..

[23] Hu X., Feng J., Zhang H., Ma J., Wu Z., Wen J., Wang S., Tian Y. (2023). Electrohydrodynamic Printing of High-Resolution Self-Reduced Soldered Silver Nanowire Pattern for Wearable Flexible Strain Sensors. Adv. Mater. Technol..

[24] Liu X., Dang A., Li T., Sun Y., Lee T.-C., Deng W., Wu S., Zada A., Zhao T., Li H. (2023). Plasmonic coupling of Au nanoclusters on a flexible MXene/Graphene oxide fiber for ultrasensitive SERS sensing. ACS Sensors.

[25] Almohammed B., Ismail A., Sali A. (2021). Electro-textile wearable antennas in wireless body area networks: Materials, antenna design, manufacturing techniques, and human body consideration—A review. Text. Res. J..

[26] Guttula R., Nandanavanam V.R., Satyanarayana V. (2021). Design and optimization of microstrip patch antenna via improved metaheuristic algorithm. Wirel. Pers. Commun..

[27] Wang T., Chen G., Zhu J., Gong H., Zhang L., Wu H. (2021). Deep understanding of impedance matching and quarter wavelength theory in electromagnetic wave absorption. J. Colloid. Interface Sci..

[28] Bird T.S. (2021). Mutual Coupling Between Antennas.

[29] Li B., Sun Q., He J., Li Y., Mao Q. (2021). An improved protection scheme of the ground electrode line based on two frequency components injection. Int. J. Electr. Power Energy Syst..

[30] Sui X., Downing J.R., Hersam M.C., Chen J. (2021). Additive manufacturing and applications of nanomaterial-based sensors. Mater. Today.

[31] Huang D., Li Z., Zeng G., Zhou C., Xue W., Gong X., Yan X., Chen S., Wang W., Cheng M. (2019). Megamerger in photocatalytic field: 2D g-C3N4 nanosheets serve as support of 0D nanomaterials for improving photocatalytic performance. Mater. Today.

[32] Garnett E., Mai L., Yang P. (2019). Introduction: 1D nanomaterials/nanowires. Chem. Rev..

[33] Zhang S., Liu C., Zhang G., Chen Y., Shang F., Xia Q., Yang W. (2021). Full review: The progress and developing trends of nanosheet-based sensing applications. Coord. Chem. Rev..

[34] Liu Q., Gao S., Zhao Y., Tao W., Yu X., Zhi M. (2021). Review of layer-by-layer self-assembly technology for fire protection of flexible polyurethane foam. J. Mater. Sci..

[35] Cao Z., Wen Q., Wang X., Yang Q., Jiang F. (2021). An Overview of the Miniaturization and Endurance for Wearable Devices. J. Internet Things.

[36] Liu C., Zhang B., Chen W., Liu W., Zhang S. (2021). Current development of wearable sensors based on nanosheets and applications. TrAC Trends Anal. Chem..

[37] Zhou T., Zhang T. (2021). Recent Progress of Nanostructured Sensing Materials from 0D to 3D: Overview of Structure–Property-Application Relationship for Gas Sensors. Small Methods.

[38] Simegnaw A.A., Malengier B., Rotich G., Tadesse M.G., Van Langenhove L. (2021). Review on the Integration of Microelectronics for E-Textile. Materials.

[39] Candan C. (2021). Proper Definition and Handling of Dirac Delta Functions [Lecture Notes]. IEEE Signal Process. Mag..

[40] Bumbudsanpharoke N., Wongphan P., Promhuad K., Leelaphiwat P., Harnkarnsujarit N. (2022). Morphology and permeability of bio-based poly (butylene adipate-co-terephthalate) (PBAT), poly (butylene succinate) (PBS) and linear low-density polyethylene (LLDPE) blend films control shelf-life of packaged bread. Food Control.

[41] Matyas J., Slobodian P., Munster L., Olejnik R., Urbánek P. (2017). Microstrip antenna from silver nanoparticles printed on a flexible polymer substrate. Mater. Today Proc..

[42] Deng Y., Li Q., Zhou Y., Qian J. (2021). Fully inkjet printing preparation of a carbon dots multichannel microfluidic paper-based sensor and its application in food additive detection. ACS Appl. Mater. Interfaces.

[43] Guo X., Hang Y., Xie Z., Wu C., Gao L., Liu C. (2017). Flexible and wearable 2.45 GHz CPW-fed antenna using inkjet-printing of silver nanoparticles on pet substrate. Microw. Opt. Technol. Lett..

[44] Jilani S.F., Rahimian A., Alfadhl Y., Alomainy A. (2018). Low-profile flexible frequency-reconfigurable millimetre-wave antenna for 5G applications. Flex. Print. Electron..

[45] Simorangkir R.B., Yang Y., Hashmi R.M., Björninen T., Esselle K.P., Ukkonen L. (2018). Polydimethylsiloxane-embedded conductive fabric: Characterization and application for realization of robust passive and active flexible wearable antennas. IEEE Access.

[46] Li Z., Sinha S.K., Treich G.M., Wang Y., Yang Q., Deshmukh A.A., Sotzing G.A., Cao Y. (2020). All-organic flexible fabric antenna for wearable electronics. J. Mater. Chem. C.

[47] Baytöre C., Zoral E.Y., Göçen C., Palandöken M., Kaya A. Coplanar flexible antenna design using conductive silver nano ink on paper substrate for wearable antenna applications. Proceedings of the 2018 28th International Conference Radioelektronika (RADIOELEKTRONIKA).

[48] Al-Adhami A., Ercelebi E. (2021). A Flexible Metamaterial Based Printed Antenna for Wearable Biomedical Applications. Sensors.

[49] Susmel L. (2022). Notches, nominal stresses, fatigue strength reduction factors and constant/variable amplitude multiaxial fatigue loading. Int. J. Fatigue.

[50] Ramli M.R., Ibrahim S., Ahmad Z., Abidin I.S.Z., Ain M.F. (2019). Stretchable conductive ink based on polysiloxane–Silver composite and its application as a frequency reconfigurable patch antenna for wearable electronics. ACS Appl. Mater. Interfaces.

[51] Zhou Y., Soltani S., Li B.M., Wu Y., Kim I., Soewardiman H., Werner D.H., Jur J.S. (2020). Direct-write spray coating of a full-duplex antenna for e-textile applications. Micromachines.

[52] Alqadami A.S., Jamlos M.F., Jamlos M.A. (2019). Efficacy of a wideband flexible antenna on a multilayer polymeric nanocomposites Fe_3_O_4_-PDMS substrate for wearable applications. Curr. Appl. Phys..

[53] Su W., Zhu J., Liao H., Tentzeris M.M. (2020). Wearable antennas for cross-body communication and human activity recognition. IEEE Access.

[54] Qiu H., Liu H., Jia X., Liu X., Li Y., Jiang T., Xiong B., Yang Y., Ren T.-l. (2018). Ink-injected dual-band antennas based on graphene flakes, carbon nanotubes and silver nanowires. RSC Adv..

[55] Jiang Z.H., Cui Z., Yue T., Zhu Y., Werner D.H. (2017). Compact, highly efficient, and fully flexible circularly polarized antenna enabled by silver nanowires for wireless body-area networks. IEEE Trans. Biomed. Circuits Syst..

[56] Wu C.-T., Ho Y.-R., Huang D.-Z., Huang J.-J.J.S., Technology C. (2019). AZO/silver nanowire stacked films deposited by RF magnetron sputtering for transparent antenna. Surf. Coat. Technol..

[57] Nair N.M., Pakkathillam J.K., Kumar K., Arunachalam K., Ray D., Swaminathan P. (2020). Printable silver nanowire and PEDOT: PSS nanocomposite ink for flexible transparent conducting applications. ACS Appl. Electron. Mater..

[58] Li W., Meredov A., Shamim A. Silver nanowire based flexible, transparent, wideband antenna for 5G band application. Proceedings of the 2019 IEEE International Symposium on Antennas and Propagation and USNC-URSI Radio Science Meeting.

[59] Dao Q., Tchuigoua R., Geck B., Manteuffel D., von Witzendorff P., Overmeyer L. Optically transparent patch antennas based on silver nanowires for mm-wave applications. Proceedings of the 2017 IEEE International Symposium on Antennas and Propagation & USNC/URSI National Radio Science Meeting.

[60] Arun H. (2021). Advancements in the use of carbon nanotubes for antenna realization. AEU-Int. J. Electron. Commun..

[61] Mansor M., Rahim S., Hashim U. A CPW-fed 2.45 GHz wearable antenna using conductive nanomaterials for on-body applications. Proceedings of the 2014 IEEE Region 10 Symposium.

[62] Behera S.K., Karmakar N.C. (2021). Chipless RFID printing technologies: A state of the art. IEEE Microw. Mag..

[63] Cheng G., Wu Y.-M., Li B. CNT-based conformal antenna design suitable for inkjet printing. Proceedings of the 2017 International Applied Computational Electromagnetics Society Symposium (ACES).

[64] Wang Y., Zhang X., Zhang X., Zhou T., Cui Z., Zhang K. (2022). A novel terahertz metasurface based on a single-walled carbon nanotube film for sensing application. J. Mater. Chem. A.

[65] Alsulami Q.A., Rajeh A. (2021). Synthesis of the SWCNTs/TiO_2_ nanostructure and its effect study on the thermal, optical, and conductivity properties of the CMC/PEO blend. Results Phys..

[66] Hajjyahya M., Ishtaiwi M., Sayyed J., Saddouq A. (2021). Design of Carbon Nanotube Antenna in Nanoscale Range. Open J. Antennas Propag..

[67] Amram Bengio E., Senic D., Taylor L.W., Headrick R.J., King M., Chen P., Little C.A., Ladbury J., Long C.J., Holloway C.L. (2019). Carbon nanotube thin film patch antennas for wireless communications. Appl. Phys. Lett..

[68] Chowdhury M.S.U., Rahman M.A., Hossain M.A., Mobashsher A.T. A transparent conductive material based circularly polarized nano-antenna for THz applications. Proceedings of the 2020 IEEE Region 10 Symposium (TENSYMP).

[69] Aissa B., Haddad E., Jamroz W., Nedil M. Design and fabrication of fluidic patch antenna based liquid metal alloy (EGaIn) and single wall carbon nanotubes nanocomposites. Proceedings of the 2013 IEEE Antennas and Propagation Society International Symposium (APSURSI).

[70] Olejník R., Goňa S., Slobodian P., Matyáš J., Moučka R., Daňová R. (2020). Polyurethane-carbon nanotubes composite dual band antenna for wearable applications. Polymers.

[71] Bayram Y., Zhou Y., Shim B.S., Xu S., Zhu J., Kotov N.A., Volakis J.L. (2010). E-textile conductors and polymer composites for conformal lightweight antennas. IEEE Trans. Antennas Propag..

[72] Li Y., Tian X., Gao S.P., Jing L., Li K., Yang H., Fu F., Lee J.Y., Guo Y.X., Ho J.S. (2020). Reversible crumpling of 2D titanium carbide (MXene) nanocoatings for stretchable electromagnetic shielding and wearable wireless communication. Adv. Funct. Mater..

[73] Tanaka H., Ohno Y., Tadokoro Y. (2016). Adaptive control of angular sensitivity for VHF-band nano-antenna using CNT mechanical resonator. IEEE Trans. Mol. Biol. Multi-Scale Commun..

[74] Mohammed K.I., Zaidan A.A., Zaidan B.B., Albahri O.S., Alsalem M.A., Albahri A.S., Hashim M. (2019). Real-time remote-health monitoring systems: A review on patients prioritisation for multiple-chronic diseases, taxonomy analysis, concerns and solution procedure. J. Med. Syst..

[75] Wang Y., Bao J., Tian Y., Wang Z., Li N. (2021). Design of High Gain Wearable Antenna Based on Wireless Body Area Network Communications. J. Phys. Conf. Ser..

[76] Kamel S., Khattab T.A. (2021). Recent advances in cellulose supported metal nanoparticles as green and sustainable catalysis for organic synthesis. Cellulose.

[77] Zavanelli N., Yeo W.-H. (2021). Advances in screen printing of conductive nanomaterials for stretchable electronics. ACS Omega.

[78] Kumar A., Shaikh M.O., Chuang C.-H. (2021). Silver nanowire synthesis and strategies for fabricating transparent conducting electrodes. Nanomaterials.

[79] Ashok Kumar S.S., Bashir S., Ramesh K., Ramesh S. (2022). A review on graphene and its derivatives as the forerunner of the two-dimensional material family for the future. J. Mater. Sci..

[80] Tallentire J. (2022). The new “Gold Rush”: Graphene’s research renaissance. Graphene.

[81] Mohanty A., Acharya O.P., Appasani B., Sooksood K., Mohapatra S.K. (2021). A THz Metamaterial Absorber with Multiple Polarization: Insensitive, Sensitive, and Tunable. ECTI Trans. Electr. Eng. Electron. Commun..

[82] Han Y.-L., Turns J., Cook K.E., Mason G.S., Shuman T.R. (2022). Students’ Experience of an Integrated Electrical Engineering and Data Acquisition Course in an Undergraduate Mechanical Engineering Curriculum. IEEE Trans. Educ..

[83] Goyal T., Majumder M.K., Kaushik B.K. (2021). Modeling and fabrication aspects of Cu-and carbon nanotube-based through-silicon vias. IETE J. Res..

[84] Dash S., Patnaik A. (2018). Material selection for TH z antennas. Microw. Opt. Technol. Lett..

[85] Atser A., Mom J., Igwue G. The Comparative Analysis of Graphene Nano-based and Copper Nano-based Patched Antenna using HFSS. Proceedings of the IOP Conference Series: Earth and Environmental Science.

[86] Abohmra A., Jilani F., Abbas H., Imran M.A., Abbasi Q.H. Terahertz antenna based on graphene for wearable applications. Proceedings of the 2019 IEEE MTT-S International Wireless Symposium (IWS).

[87] Tang D., Wang Q., Wang Z., Liu Q., Zhang B., He D., Wu Z., Mu S. (2018). Highly sensitive wearable sensor based on a flexible multi-layer graphene film antenna. Sci. Bull..

[88] Fang R., Song R., Zhao X., Wang Z., Qian W., He D. (2020). Compact and low-profile UWB antenna based on graphene-assembled films for wearable applications. Sensors.

[89] Wang C., Song R., Jiang S., Hu Z., He D. (2022). Low profile and miniaturized dual-band antenna based on graphene assembled film for wearable applications. Int. J. RF Microw. Comput.-Aided Eng..

[90] Zhang J., Song R., Zhao X., Fang R., Zhang B., Qian W., Zhang J., Liu C., He D. (2020). Flexible graphene-assembled film-based antenna for wireless wearable sensor with miniaturized size and high sensitivity. ACS omega..

[91] Li S., Song R., Zhang B., Huang B., Zhao X., He D. (2021). Wearable near-field communication bracelet based on highly conductive graphene-assembled films. Int. J. RF Microw. Comput.-Aided Eng..

[92] Zu H.-R., Wu B., Zhang Y.-H., Zhao Y.-T., Song R.-G., He D.-P. (2020). Circularly polarized wearable antenna with low profile and low specific absorption rate using highly conductive graphene film. IEEE Antennas Wirel. Propag. Lett..

[93] Ergoktas M.S., Bakan G., Steiner P., Bartlam C., Malevich Y., Ozden-Yenigun E., He G., Karim N., Cataldi P., Bissett M.A. (2020). Graphene-enabled adaptive infrared textiles. Nano Lett..

[94] Ibanez-Labiano I., Ergoktas M.S., Kocabas C., Toomey A., Alomainy A., Ozden-Yenigun E. (2020). Graphene-based soft wearable antennas. Appl. Mater. Today.

[95] Bala R., Singh R., Marwaha A., Marwaha S. (2016). Wearable graphene based curved patch antenna for medical telemetry applications. Appl. Comput. Electromagn. Soc. J. (ACES).

[96] Ali U., Basir A., Zada M., Ullah S., Kamal B., Yoo H. (2023). Performance Improvement of a Dual-Band Textile Antenna for On-Body Through Artificial Magnetic Conductor. IEEE Access.

[97] Akbari M., Virkki J., Sydänheimo L., Ukkonen L. (2016). Toward graphene-based passive UHF RFID textile tags: A reliability study. IEEE Trans. Device Mater. Reliab..

[98] Kapetanakis T.N., Nikolopoulos C.D., Petridis K., Vardiambasis I.O. (2021). Wearable textile antenna with a graphene sheet or conductive fabric patch for the 2.45 GHz band. Electronics.

[99] Német A., Alkaraki S., Abassi Q.H., Jilani S.F. A Biodegradable Textile-based Graphene Antenna for 5G Wearable Applications. Proceedings of the 2021 IEEE International Symposium on Antennas and Propagation and USNC-URSI Radio Science Meeting (APS/URSI).

[100] Labiano I.I., Jilani S.F., Ergoktas M.S., Kocabas C., Ozden-Yenigun E., Alomainy A. Graphene-based Textile Ultra Wideband Antennas for Integrated and Wearable Applications. Proceedings of the 2019 IEEE International Symposium on Antennas and Propagation and USNC-URSI Radio Science Meeting.

[101] Huang X., Leng T., Zhang X., Chen J.C., Chang K.H., Geim A.K., Novoselov K.S., Hu Z. (2015). Binder-free highly conductive graphene laminate for low cost printed radio frequency applications. Applied Physics Letters..

[102] Leng T., Huang X., Chang K., Chen J., Abdalla M.A., Hu Z. (2016). Graphene nanoflakes printed flexible meandered-line dipole antenna on paper substrate for low-cost RFID and sensing applications. IEEE Antennas Wirel. Propag. Lett..

[103] Huang X., Leng T., Zhu M., Zhang X., Chen J., Chang K., Aqeeli M., Geim A.K., Novoselov K.S., Hu Z. (2015). Highly flexible and conductive printed graphene for wireless wearable communications applications. Sci. Rep..

[104] Lamminen A., Arapov K., de With G., Haque S., Sandberg H.G., Friedrich H., Ermolov V. (2017). Graphene-flakes printed wideband elliptical dipole antenna for low-cost wireless communications applications. IEEE Antennas Wirel. Propag. Lett..

[105] Hu G., Kang J., Ng L.W., Zhu X., Howe R.C., Jones C.G., Hersam M.C., Hasan T. (2018). Functional inks and printing of two-dimensional materials. Chem. Soc. Rev..

[106] Tung T.T., Chen S.J., Fumeaux C., Kim T., Losic D. (2021). N-doped reduced graphene oxide-PEDOT nanocomposites for implementation of a flexible wideband antenna for wearable wireless communication applications. Nanotechnology.

[107] Salman A.R., Ismail M.M., Abd Razak J., Ab Rashid S.R. (2022). Design of UTeM logo-shape wearable antenna for communication application by graphene silver nanocomposites. TELKOMNIKA.

[108] Song R., Mao B., Wang Z., Hui Y., Zhang N., Fang R., Zhang J., Wu Y., Ge Q., Novoselov K.S. (2023). Comparison of copper and graphene-assembled films in 5G wireless communication and THz electromagnetic-interference shielding. Proc. Natl. Acad. Sci. USA.

[109] Morales-Centla N., Torrealba-Melendez R., Tamariz-Flores E.I., López-López M., Arriaga-Arriaga C.A., Munoz-Pacheco J.M., Gonzalez-Diaz V.R. (2022). Dual-Band CPW Graphene Antenna for Smart Cities and IoT Applications. Sensors.

[110] Zhang S., Zeng J., Wang C., Feng L., Song Z., Zhao W., Wang Q., Liu C. (2021). The application of wearable glucose sensors in point-of-care testing. Front. Bioeng. Biotechnol..

[111] Khodabandehlo A., Noori A., Rahmanifar M.S., El-Kady M.F., Kaner R.B., Mousavi M.F. (2022). Laser-Scribed Graphene–Polyaniline Microsupercapacitor for Internet-of-Things Applications. Adv. Funct. Mater..

